# Limb-sparing surgery as an alternative for limb amputation in an invasive myxoid liposarcoma – case report

**Published:** 2015

**Authors:** MO Adameșteanu, V Enache, D Zamfirescu, I Lascăr

**Affiliations:** * Clinic of Plastic Surgery and Reconstructive Microsurgery, Emergency Hospital Bucharest; Department of Plastic, Aesthetic Surgery and Reconstructive Microsurgery, „Carol Davila” University of Medicine and Pharmacy, Bucharest, Romania; ** Department of Pathology, Emergency Hospital Bucharest, Romania

**Keywords:** sarcomas, radical excision, limb-sparing surgery, calf, preoperative radiotherapy

## Abstract

In medical practice, plastic surgeons confront with patients with sarcomas of the extremities that require a radical surgical approach. Knowing when to attempt limb-sparing surgery and when to give in to limb amputation is one of the most difficult decisions a surgeon can take.

The correct approach and management of such cases ensure surgical success and the patient survival.

In this paper, the case of a 56-year-old man, admitted in our clinic with a crush injury of the right calf and subsequent haematoma is presented. During haematoma drainage, the surgeon noticed abnormal tissue and performed an incisional biopsy. The patient was diagnosed with myxoid liposarcoma of the external compartment of the right calf. Limb amputation was proposed, but the patient refused. After the clinical examination, blood tests and diagnostic imaging, which allowed the correct evaluation of the case-tumor sizes and neighboring tissue reports, and preoperatory radiotherapy, limb sparing surgery, respectively primary tumor excision was decided to be performed. Negative margins could not be obtained by 3 successive resections or by adjuvant chemotherapy.

The presented case supports the idea that limb-sparing surgery is only applicable to carefully selected patients with soft tissue sarcoma. In some cases, radical excision involving even mutilating amputations may provide a better oncologic and functional result.

**Abbreviations:** HE = haematoma, MRI = magnetic resonance imaging, NCCN = National Comprehensive Cancer Network, CT-SCAN = computer tomography scan, PET-CT = positron emission computer tomography, FDG = fluoro-deoxy-glucose, SUV = standardized uptake value, AJCC = American Joint Committee on Cancer

## Introduction

Soft tissue sarcomas are rare malignancies that arise from mesenchymal pluripotent stem cells, of which liposarcomas are the most common. Myxoid liposarcomas account for about 30-50% of all liposarcomas [**1**]. It involves balanced translocations (t12;16)(q13;p11), but additional chromosomal aberrations also occur [**2**-**4**]. It has a peak incidence during the fifth decade. It develops preferentially in the lower extremity (75%), in the medial thigh or popliteal region. They are generally well circumscribed but in particular cases, can interdigitate with muscles, tendons or can compress neurovascular structures. Typically, they appear as nonhomogeneous masses on CT scans [**3**]. Complete surgical resection is the only curative means for the localized disease; however, both radiation and conventional cytotoxic chemotherapy remain controversial for metastatic or unresectable disease [**4**,**5**]. Their most frequent metastases are found in lungs or bone [**6**].

## Case report

The case of a 56-year-old male patient, aged 56, polyallergic, known with high blood pressure, admitted in our clinic for a crush injury of the anterolateral aspect of the right calf and subsequent haematoma, is presented. The patient had been complaining for a discomfort to the anterior right calf during effort for the past 10 years. Four years ago, the discomfort became pain, accompanied by muscle weakness, and the patient noticed a slowly growing 5-6 cm soft tissue mass on the inferior pole of the tibialis anterior muscle. He seeked for medical attention, underwent a plain MRI investigation of the right lower limb that revealed a deep haematoma of the external aspect of the right calf 16.5/3.8/6.8cm that pierced the fascia entering the anterior compartment, with associated muscle atrophy, but without muscle invasion. The attending general surgeon diagnosed him with chronic venous insufficiency and prescribed medical treatment. After an accidental crush injury to the affected region, the patient was referred to our clinic. No medical documents were presented at admission.

Physical examination showed a global enlargement of the right calf, more significant on the anterolateral compartment, measuring 27/ 10 cm, tender, with associated muscle weakness (M3), fixed to adjacent structures. After a blood test, EKG and a cardiologic evaluation, the patient was operated on. The haematoma was evacuated through an S-shaped incision on the external aspect of the right calf. Because of the abnormal gross anatomy, the surgeon also performed an incisional biopsy. The wound was sutured, dressed and the patient discharged after 3 days.

**Fig. 1 A,B:**
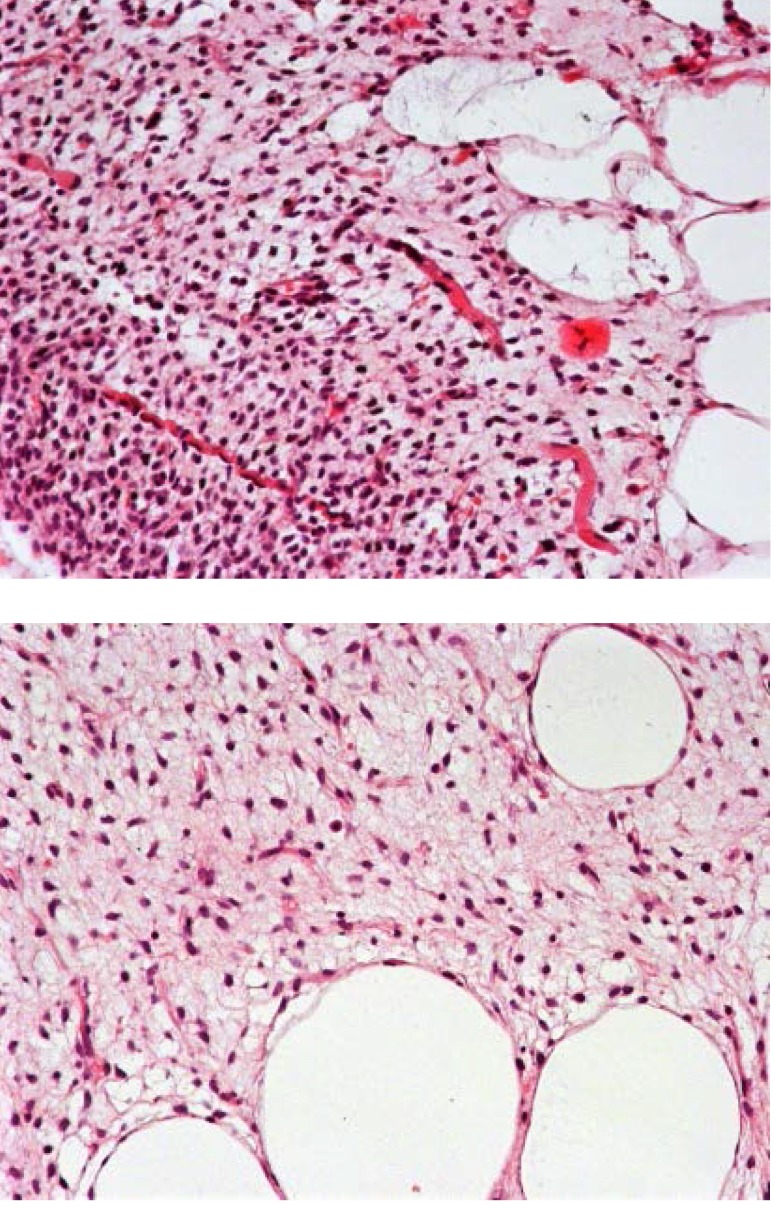
HE (hematoxilin-eosin) 200X low-grade myxoid liposarcoma showing focal areas of increased cellularity and muscle fibers [**5**]

The pathologic examination described a low-grade myxoid liposarcoma showing multiple transition areas to round cell liposarcoma G1 with positive margins and subsequent muscle fiber invasion (**Fig. 1 A,B**).

**Fig. 2 A,B:**
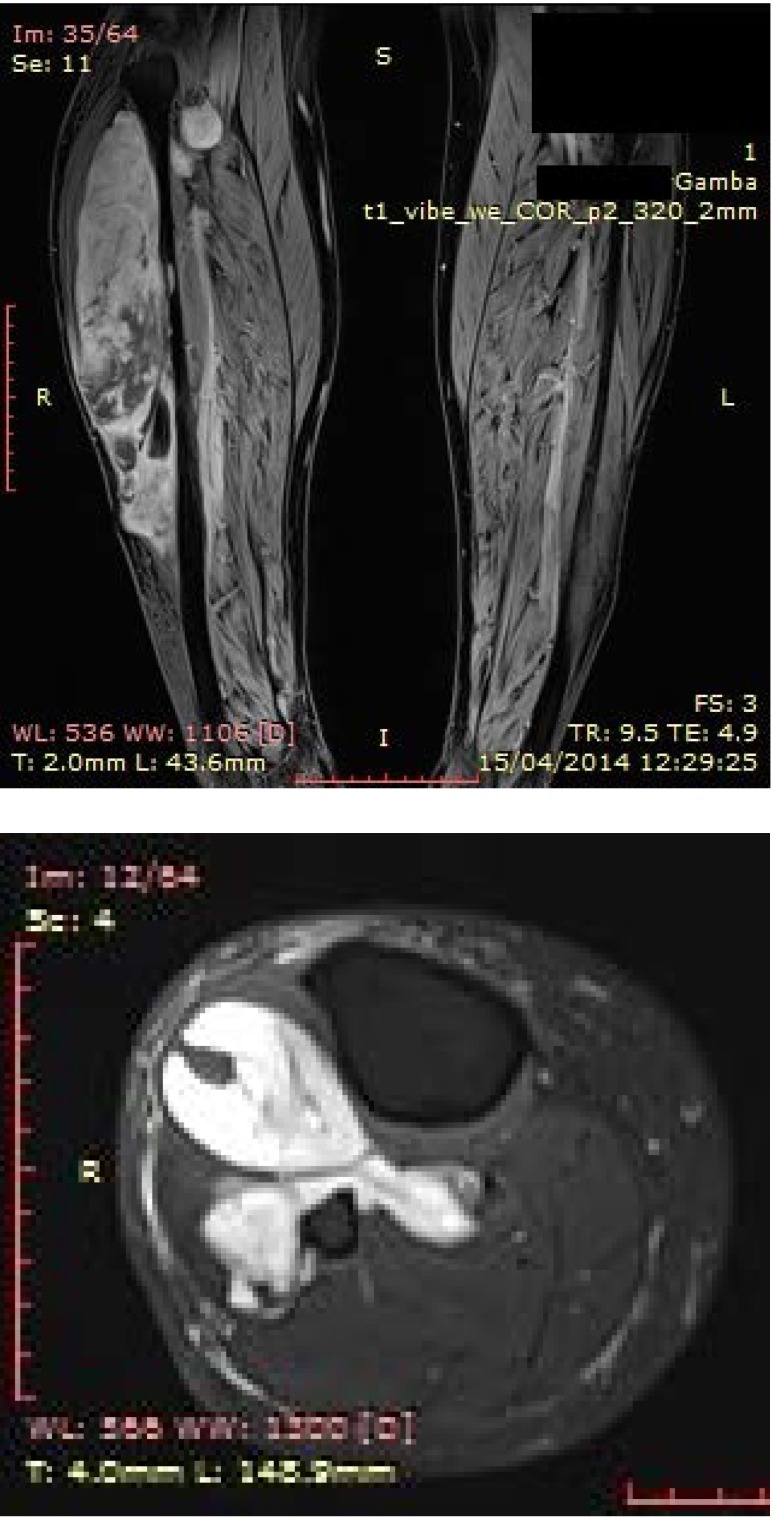
MRI- study: Soft tissue mass on the anterolateral aspect of the right calf, subsequent intratumoral necrosis, posterior compartment invasion (Personal photographic archive)

The patient was referred to an oncologist and radiation therapy specialist. The imagistic evaluation of the tumor was recommended. Gadolinum enhanced magnetic resonance imaging (MRI) of the lower right limb showed a soft tissue mass, deep in the anterior compartment of 28.6/9.4/6.1cm, with increased intake, increased vascularisation, of lipomatous signal and central necrosis, invading the tibialis posterior muscle, and compressing the fibula (**Fig. 2 A,B**).

**Fig. 3 A,B:**
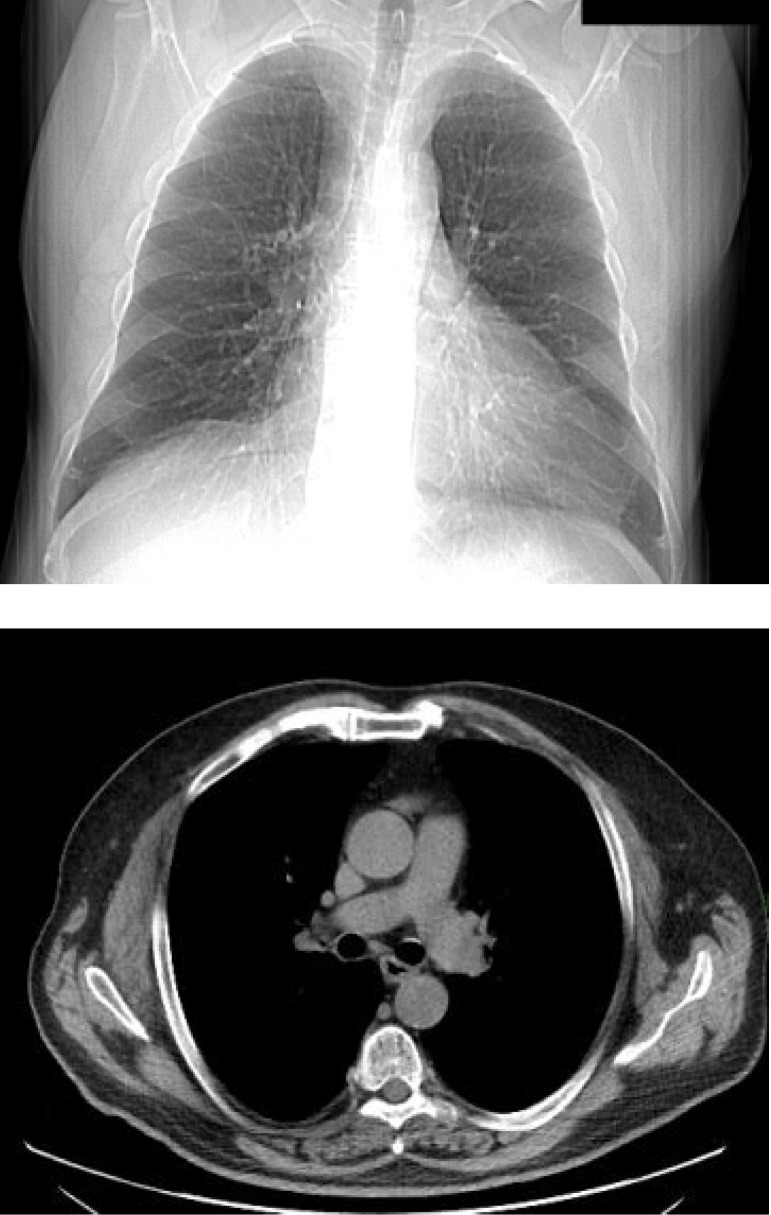
CT-scan of the thorax reveals no pathologic masses (Personal photographic archive)

Computer tomography (CT) scan of the thorax, abdomen and pelvis revealed 2 right pulmonary nodules of 5/ 4mm in the superior right lobe and 8/ 6 mm in the inferior right lobe, without distant metastases. A benign nodule of the latissimus dorsi of 14/ 7 mm was discovered (**Fig. 3 A,B**).

**Fig. 4 A,B:**
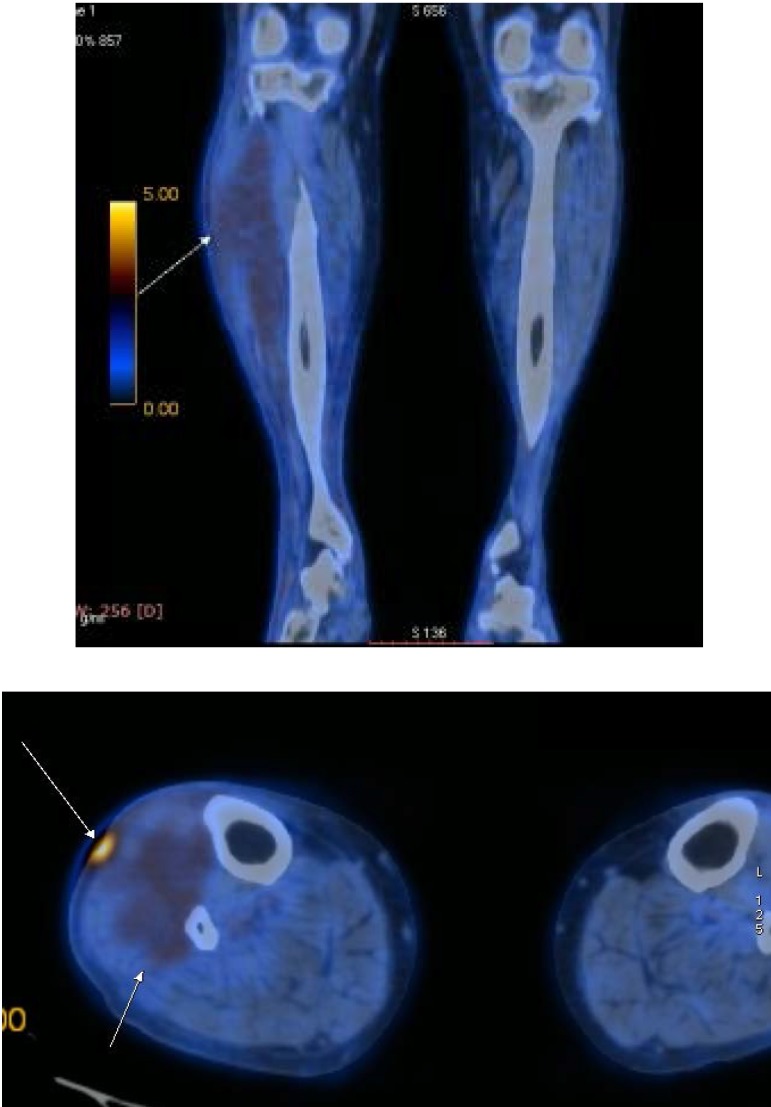
Increased intake of 18-FDG on the external compartment of the right calf (Personal photographic archive)

Whole body positron emission computer tomography (PET-CT) confirmed the presence of a benign nodule of the latissimus dorsi and increased FDG intake in the upper two-thirds of the right calf on the anterolateral compartment (standardized uptake value SUV=2,60) and subsequent skin ulceration (SUV 5,23) (**Fig. 4 A,B**).

**Fig. 5:**
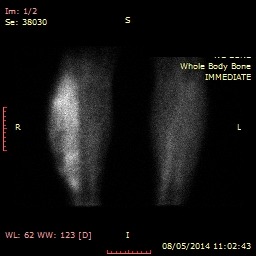
Bone scan of the right calf (Personal photographic archive)

Whole body bone scan excluded distant metastasis, showing a soft tissue mass with increased intake and high vascularisation (**Fig. 5**).

**Fig. 6:**
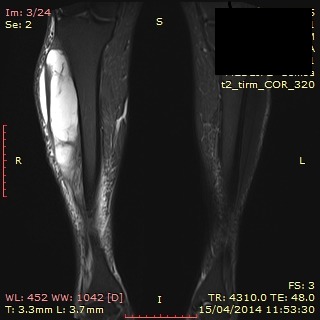
Gadolinum enhanced MRI showing significant tumor reduction (Personal photographic archive)

After a complete evaluation, the tumor was staged according to the American Joint Committee on Cancer (AJCC) system stage T2bN0M0 G1, stage IIB disease. Due to posterior and anterolateral compartment involvement, limb-sparing surgery could not provide the necessary margins of safety. The radical surgical procedure was imposed by the tumor’s large dimension, muscle invasion and invasion of vascular and nervous bundle. Amputation was proposed, but refused by the patient. In order to perform the radical excision of the tumor, radiation therapy was necessary. Neoadjuvant external beam radiation therapy was administered using a regimen of 2Gy in 25 fractions in 30 days. The MRI examination showed a significant decrease in tumor size to 18/7,5/5.6 cm, with no posterior compartment invasion. Unfortunately, fibular invasion and involvement of the tibialis posterior artery and were noted. An area of skin invasion of 2.5 cm was visible (**Fig. 6**).

**Fig. 7:**
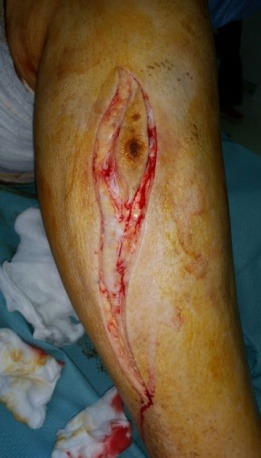
Intraoperatory aspects (Personal photographic archive)

**Fig. 8:**
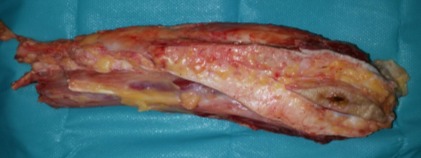
Resected tumor (Personal photographic archive)

 The patient was admitted in our Clinic, and after a preoperatory workup, an S-shaped incision was placed on the external aspect of the right calf, en bloc excision of the primary tumor, drainage, suture and splinting were performed (**Fig. 7**). The resected piece, consisted in a 6/ 2 cm skin paddle, 12 cm of fibula, tibialis anterior, extensor hallucis, extensor digitorum, peronier, tibialis posterior and external half of the soleus muscles were sent for pathologic examination (**Fig. 8**). All the margins were positive.

**Fig. 9:**
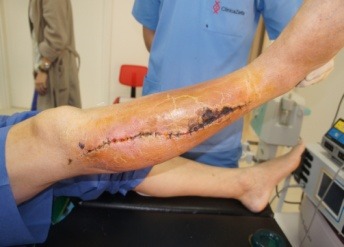
Skin necrosis (Personal photographic archive)

The patient was discharged after 3 days and developed a 5/ 4cm necrosis on the anterolateral aspect of the right calf, that was surgically removed, en-bloc with a 3 cm mass of soleus muscle (**Fig. 9**). The excision margins were positive. 

The patient was referred to the oncologist, and is currently undergoing a chemotherapy regimen with Ifosfamide 5 g/ m2 and Doxorubicin 50 mg/ m2 once a week and a pulse of 20Gy of radiation therapy to the tumor bed. The disease specific survival probability at 5 years, in this case, is 80%, and 63% for 12 years. Clinical and imagistic evaluation at each 3, 6, 12, 18, 24, 36 months postoperatory is mandatory, then yearly the next 2 years.

## Discussion

A case of myxoid liposarcoma in a 56-year-old man, presenting as a haematoma, was reported. After complex investigations and interdisciplinary involvement (plastic surgeon, oncologist, radiation therapist) the final treatment plan was established. After the patient’s refusal of the amputation, the surgical plan was adapted to the patient’s wish. The oncologic treatment was mandatory. The prognosis was uncertain, most probably, the patient already having suffered from the metastatic spread, even if it was not observed on the CT scans. All the efforts having been undertaken, the patient’s overall survival and disease free interval remained to be observed.

Case particularities included misdiagnosis of the tumor due to plain MRI, traumatic injury of the soft tissue sarcoma causing inadvertent biopsy, the NCCN 2014 (National Comprehensive Cancer Network) guideline-oriented approach, the refusal of the proposed amputation, the administration of neoadjuvant radiation therapy and the postoperatory association of chemotherapy and radiation therapy.

The following conclusions can be drawn: 

• Soft tissue sarcomas are frequently misdiagnosed, due to improper clinical or imagistic investigations.

• Preoperatory imagistic evaluation by gadolinium enhanced MRI represents the current golden standard evaluating the tumor’s extension both at surface and in depth, allowing the surgeon to accurately plan the radical excision.

• The surgical treatment is the radical excision of the primary tumor with negative margins. This supposes, quite often, the neighboring muscle resection or total amputation. Whenever possible, limb-sparing surgery should be performed. 

• Even low-grade tumors such as myxoid liposarcoma can be invasive and may require limb amputation.

• Patient refusal of the correct therapy, forced the surgeon to perform the incomplete tumor resection.

• Repeated reinterventions with persisting positive margins cause significant morbidity and decrease overall survival rates, proving that limb sparing should never prevail to radicality.

**Acknowledgments**

This paper was co-financed from the European Social Fund, through the Sectorial Operational Programme Human Resources Development 2007-2013, project number POSDRU/159/1.5/S/138907 “Excellence in scientific interdisciplinary research, doctoral and postdoctoral, in the economic, social and medical fields –EXCELIS”, coordinator The Bucharest University of Economic Studies”. 

**Disclosures:** None

## References

[1] Daniels J, Green C, Paul A (2014). Liposarcoma of the Great Toe: A Case Report. The Journal of Foot and Ankle Surgery.

[2] (2008). Encyclopedia of Genetics, Genomics, Proteomics and Informatics.

[3] Enziger FM, Weiss SW (1995). Soft tissue sarcoma.

[4] Guan Z, Yu X, Wang H, Zhang J, Cao J, Li G, Teng L (2015). Advances in the targeted therapy of liposarcoma. Onco Targets Ther.

[5] Kollar A, Benson C (2014). Current management options for liposarcoma and challenges for the future. Expert Rev Anticancer Ther.

[6] Schwab J, Boland P, Guo T, Brennan M, Singer S, Healey J, Antonescu C (2007). Skeletal Metastases in Myxoid Liposarcoma: An Unusual Pattern of Distant Spread. Annals of Surgical Oncology.

